# CRISPR Cas9-mediated ablation of pyruvate carboxylase gene in colon cancer cell line HT-29 inhibits growth and migration, induces apoptosis and increases sensitivity to 5-fluorouracil and glutaminase inhibitor

**DOI:** 10.3389/fonc.2022.966089

**Published:** 2022-11-10

**Authors:** Jarunya Ngamkham, Siraprapa Siritutsoontorn, Saowaluk Saisomboon, Kulthida Vaeteewoottacharn, Sarawut Jitrapakdee

**Affiliations:** ^1^ Graduate Program in Molecular Medicine, Faculty of Science, Mahidol University, Bangkok, Thailand; ^2^ Department of Biochemistry, Faculty of Science, Mahidol University, Bangkok, Thailand; ^3^ Department of Biochemistry, Faculty of Medicine, Khon Kaen University, Khon Kaen, Thailand

**Keywords:** pyruvate carboxylase, cancer metabolism, colorectal cancer, 5-FU, glutaminase

## Abstract

Pyruvate carboxylase (PC) is an important anaplerotic enzyme that replenishes the tricarboxylic acid cycle (TCA) intermediates. It prevents the collapse of the TCA cycle upon its intermediates are removed during high anabolic demand. We have recently shown that overexpression of PC protein was associated with staging, metastasis and poor survival of colorectal cancer patients. Herein, we generated the PC knockout (PC KO) colon cancer cell lines, HT-29, by CRISPR-Cas9 technique, as a model to understand the role of this enzyme in colorectal cancer. The PC KO HT-29 cell lines had no detectable PC protein and did not show abnormal cellular or nuclear structures. However, PC KO HT-29 cells showed a 50-60% reduction in their growth rate and a 60-70% reduction in migration. The deficient growth phenotype of PC KO HT-29 cells was associated with apoptotic induction with no apparent cell cycle disruption following five days of growth. Down-regulation of key lipogenic enzymes, including acetyl-CoA carboxylase-1 and fatty acid synthase, was also associated with growth inhibition, suggesting that the *de novo* lipogenesis is impaired. Furthermore, PC KO HT-29 cells were 50% and 60% more sensitive to 5-fluorouracil and glutaminase inhibitor, CB-839, at their IC_50_ concentrations, respectively, following 48 h exposure. The increased cytotoxicity of CB-839 to PC KO HT-29 cells was associated with increased poly (ADP-ribose) polymerase cleavage. However, this was not observed with PC KO cells exposed to 5-fluorouracil, suggesting that PC KO HT-29 cells were prone to CB-839-induced apoptosis. Collectively, these findings indicate that ablation of PC expression in HT-29 cells disrupts the metabolic homeostasis of cells and inhibits proliferation and migration, accompanied by apoptotic induction. This study highlights the crucial role of PC in supporting the survival of HT-29 cells during exposure to chemotherapeutic drugs.

## Introduction

Colorectal cancer (CRC) is the third most frequent cancer and the second leading cause of cancer-associated death worldwide. CRC mainly affects adults who are >50 years old (1). Similar to other types of cancer, metastasis is the primary cause of death in patients with colorectal carcinoma. The most common metastatic site for CRC is the liver, but it may also invade other distal organs such as the lung, bone and brain (2). Like other types of cancers, increasing proliferative signals, evading growth suppression, increasing cellular motility and invasion, escaping immune surveillance, enabling replicative immortality, resisting apoptosis and altering cellular metabolism are the hallmarks of CRC (3, 4). Targeting cancer metabolism has received much attention in the past decades because most cancers reprogram their metabolisms such as increased aerobic glycolysis and pentose phosphate pathway, increased *de novo* lipogenesis and nucleotide biosynthesis to support their biomass and bioenergetic demands (5, 6). The discovery of small molecules that can inhibit the activity of key enzymes of the above metabolic pathways has paved the way for the new anti-cancer drugs (7–9).

The tricarboxylic acid (TCA) cycle is not only served as an oxidative hub for acetyl-CoA generated from glucose and fatty acid catabolism but also functions as an anabolic hub for the synthesis of amino acids, nucleotides and fatty acids (10). A rapid removal of TCA cycle intermediates during high anabolic demand can perturb TCA cycle activity and impair both oxidative and biosynthetic fates of the TCA cycle. The maintenance of TCA cycle intermediates requires anaplerosis, which replenishes its intermediates upon their rapid removal for biosynthesis (10). In cancers, two major anaplerotic reactions exist, including glutaminolysis and pyruvate carboxylation. The former reaction is the conversion of glutamine to α-ketoglutarate by two enzymes, i.e., glutaminase (GLS) and glutamate dehydrogenase, respectively (11). In contrast, pyruvate carboxylation is the carboxylation of pyruvate to oxaloacetate, catalyzed by the pyruvate carboxylase (PC) (12, 13). Depending on genetic background and availability of nutrients, some cancers such as non-small cell lung cancer (14, 15), breast cancer (16), succinate dehydrogenase-deficient paraganglioma cancer (17) rely on pyruvate carboxylation while glioblastoma or thyroid cancer can switch between these two reactions, depending on glutamine availability (18, 19). Overexpression of PC or GLS in the above tumors reflects their role in supporting cellular metabolism. Recently, we have shown that PC protein is also overexpressed in colorectal tissue of CRC patients, in which a high level of PC was associated with advanced tumor stage, perineural invasion, lymph node metastasis, and poor prognosis (20). The co-elevation of transcript for the holocarboxylase synthetase, an enzyme that biotinylates PC in colorectal tissue of CRC patients, also further suggests the crucial role of PC in supporting the growth and invasion of CRC (20, 21). Although the above study provides a tight association between high PC expression and clinicopathological parameters, there was no direct evidence regarding the pro-oncogenic role of PC in supporting the growth and survival of CRC. Here, we show that ablation of the PC gene in HT-29 colorectal cancer by CRISPR-Cas 9 gene editing inhibited proliferation, migration and increased sensitivity to 5-fluorouracil (5-FU) or CB-389, leading to apoptosis.

## Materials and methods

### Cell lines and maintenance

Human colorectal adenocarcinoma, HT-29 cell line (ATCC HTB-38™) was cultured in Dulbecco’s modified Eagle medium/F12 (1:1) (DMEM/F12) (Invitrogen) supplemented with 10% (v/v) fetal bovine serum (Invitrogen) and 100 units/ml penicillin, 100 µg/ml streptomycin (Invitrogen). Cells were then maintained in a humidified incubator at 37°C with 5% CO_2_. Cells were grown to 60-80% confluence before sub-culturing at the ratio of 1:5. CRISPR Cas9-mediated PC KO HT-29 and HT-29 cells transfected with empty vector (control cell line) were generated as described previously (20). Following green fluorescent protein sorting, three PC KO cell lines, PC-gRNA1, PC-gRNA2, and PC-gRNA3 were expanded and cultured in the minimal essential medium for proliferation and apoptotic assays.

### MTS Assay

Cell viability was determined using an MTS reagent (Promega). Briefly, 5x10^3^ cells/well were seeded in 96-well microplates containing minimal essential medium (MEM) supplement with 10% fetal bovine serum and 1% penicillin-streptomycin. After 1, 2, 3, 4, 5, 6, and 7 days, the cultured medium was replaced with 100 µl of fresh medium containing 20 µl of CellTiter One solution (MTS solution) before incubated at 37°C, 5% CO_2_ for 1 h. The amounts of soluble formazan were measured at an absorbance of 540 nm. The results are expressed as means with standard derivations of three independent experiments. For restoration of growth deficiency by aspartate, 5 x 10^4^ cells of parental HT-29 or PC KO HT-29 cells were plated in the above complete MEM and maintained at 37°C for 24 h. On the next day, fresh medium without or with 10 mM aspartate was replaced, and cultures were maintained for 48 h before viability was assessed using MTS reagent as described above.

### Migration assay

Migration was analyzed using Boyden chamber assay with transwell cell culture inserts (Corning Incorporated, Corning, NY). 1.5x10^5^ cells in 200 µl of serum-free DMEM medium were seeded into the upper chamber of the transwell and allowed to migrate to the lower chamber containing 600 µl of DMEM containing 20% (v/v) fetal bovine serum at 37°C for 48 h. Migrated cells were fixed with 4% (v/v) formaldehyde and stained with 0.1% (w/v) crystal violet. Five random fields were counted under a microscope and expressed as a percentage of migration relative to the control cell line. Experiments were performed in duplicate, and the data presented are the average from three independent experiments.

### Apoptosis assay

Apoptosis was detected using Alexa Fluor 488 Annexin V/Dead Cell Apoptosis Kit (ThermoFisher, CA, USA). 2.5 x10^5^ cells were cultured in 35-mm^2^ dish containing MEM with 10% (v/v) fetal bovine serum. Both detached and adherent cells were collected from cultured dishes by centrifugation at 3,000 rpm for 5 min, and the supernatant was discarded. The cell pellet was suspended in 100 μl of 1x annexin-binding buffer before adding 5 μl of annexin V and 100 μg/ml of PI solution. The samples were mixed gently and incubated at room temperature for 15 min in the dark. Four hundred microlitres of 1x annexin-binding buffer were added to suspension cells and kept on ice before analysis by flow cytometer (Attune NxT flow cytometer, ThermoFisher, USA). Apoptotic profiles of cells were analyzed using InvitrogenTM Attune NxT Software (ThermoFisher, USA).

### Drug sensitivity assay

5x10^3^ cells of parental HT-29 or HT-29 transfected with an empty vector were seeded in a 6-well plate containing MEM medium supplement with 10% (v/v) fetal bovine serum and 1% penicillin-streptomycin. Following 24 h culture, the old medium was replaced with a fresh medium containing 0-7.7 mM of 5-FU (Sigma-Aldrich, St Louis, USA) or 0-1000 µM of CB-839 (MedChem Express (Monmouth Junction, NJ, USA) at 37°C for 48 h. Stock solutions of 5-FU and CB-839 were prepared by dissolving the drug in 0.2% (v/v) DMSO before being 10-fold diluted in a culture medium. The viability of cells exposed to different drug concentrations was determined by an MTS assay. Dose-response curve of the drug was plotted between absorbance at 540 nm and drug concentrations. Half-maximal inhibitory concentration (IC_50_) was then calculated from the graph. The viability of PC KO HT-29 cells cultured in the absence or presence of drug was relative to that of the empty vector transfected-HT-29 cells (control cell line) cultured in MEM without drug, which was arbitrarily set to 100%. DMSO was also included in MEM at a final concentration of 0.02% (v/v) (ThermoFisher, USA) for the no-drug treatment group. Apoptotic profiles of cells were analyzed using InvitrogenTM Attune NxT Software (ThermoFisher, USA).

### RNA isolation and quantitative real time-PCR (qRT-PCR)

Total RNA was extracted using TRIzol reagent (Gibco). 2 µg of total RNA were primed with 0.2 µg of random hexamers in a 10 µl mixture before incubating at 70°C for 5 min. Reverse transcription was carried out in a 20 µl-reaction mixture containing 1xImProm-IITM reaction buffer, 3 mM MgCl_2_, 0.5 mM dNTP and 160 units of ImProm-II reverse transcriptase enzyme (Promega) and incubated at 25°C for 5 min, 42°C for 60 min and 70°C for 15 min, respectively. Quantitative real-time PCR was performed in a 12 µl-reaction mixture containing 2x KAPA probe FastqPCR master mix Universal (KAPA Biosystem), 2 µl of cDNA, 1 µM each of human PC primers (5’-GATGACTTCACAGCCCAG-3’ and 5’-GGGCACCTCTGTGTGCAG-3’) or 18s ribosomal RNA primers (5’-CGGCTACCACATCCAAGGAA-3’ and 5’-GCTGGAATTACCGCGGCT-3’) and 0.5 µM of fluorogenic probes for PC (5’-FAM-CCCTGGTGGCCTGTACCAAAGGG-TAMRA-3’) and 18s rRNA (5’-VIC-TGCTGGCACCAGACTTGCCCTC-TAMRA-3’), respectively. qRT-PCR of ACC1 and FASN mRNA expression was similarly performed in a 12 µl-reaction mixture but with 1x KAPA SYBR FAST qPCR Master mix universal (KAPA Biosystem) with primers specific for human ACC1 (5’-GCTCCTTGTCACCTGCTTCT-3’ and 5’- CAAGGCCAAGCATCCTGTA -3’) or human FASN (5’-CTTCCGAGATTCCATCCTACGC-3’ and 5’- TGGCAGTCAGGCTCACAAACG -3’). The PCR profiles consisted of an initial incubation at 50°C for 2 min and 95°C for 10 min, followed by 40 cycles of denaturation at 95°C for 15 sec and annealing/extension at 60°C for 1 min using Mx3000P Q-PCR system (Agilent Technologies). The expression of PC mRNA was normalized with that of 18s rRNA and shown as the relative gene expression. Fold change of gene expression was calculated using the comparative cycle threshold ( ΔΔCt) method (22).

### SDS-PAGE and Western blot analysis

Monolayer cell obtained from 35-mm^2^ culture dish was lysed in in 50 µl of radio-immuno-precipitation assay buffer containing 50 mM Tris-HCl pH 7.4, 150 mM NaCl, 1 mM EDTA, 0.25% (w/v) sodium deoxycholate, 1% (v/v) NP-40, 1 mM DTT and 1x protease inhibitor cocktail (Roche, Basel, Switzerland). Protein concentrations in cell lysates were determined using the Bradford reagent (BioRad). Twenty micrograms of whole-cell lysates were analyzed by 6.5% or 7.5% discontinuous SDS-polyacrylamide gel electrophoresis using 1x glycine buffer (25mM Tris-HCl pH 8.3, 193 mM glycine, 0.1% (w/v) SDS) (23). Proteins were transferred to polyvinylidene difluoride membrane using Semi-Dry Transfer Cell (Bio-Rad) at constant 12 volts for 90 min. Membranes were incubated in 5% (w/v) skim milk or 3% (w/v) BSA in 1x TBS-T (50mM Tris-HCl pH 7.6, 150mM NaCl and 0.1% Tween 20) room temperature or at 4°C overnight. The membrane was incubated with different primary antibodies, i.e., 1:20,000 dilution of rabbit anti-PC polyclonal antibody (16), 1:10,000 dilution of mouse anti-ACC1 monoclonal antibody (67373-1-lg; Proteintech group, IL, USA), 1:10,000 dilution of mouse anti-FASN monoclonal antibody (66591-1-lg, Proteintech group, IL, USA), 1:1,000 dilution of rabbit anti-PARP polyclonal antibody (9542; Cell Signaling Technology), 1:10,000 dilution of mouse anti-β actin monoclonal antibody (A1978; Sigma) overnight. The blots were washed with 1x TBS-T three times before incubating 1:10,000 dilution of goat anti-rabbit (P044; DAKO, CA, USA) or goat anti-mouse IgG antibody (31430; Invitrogen, IL, USA) conjugated with horseradish peroxidase for 1 h at room temperature. The secondary antibody was removed by washing with 1x TBS-T three times. The chemiluminescence bands were detected using a chemiluminescence HRP detection reagent (Millipore, Burlington, MA, USA), and images were captured using an enhanced chemiluminescence imaging system (Syngene, Frederick, MA, USA).

## Results

### PC KO HT-29 cells showed impaired cell growth and migration

**Figure 1:**
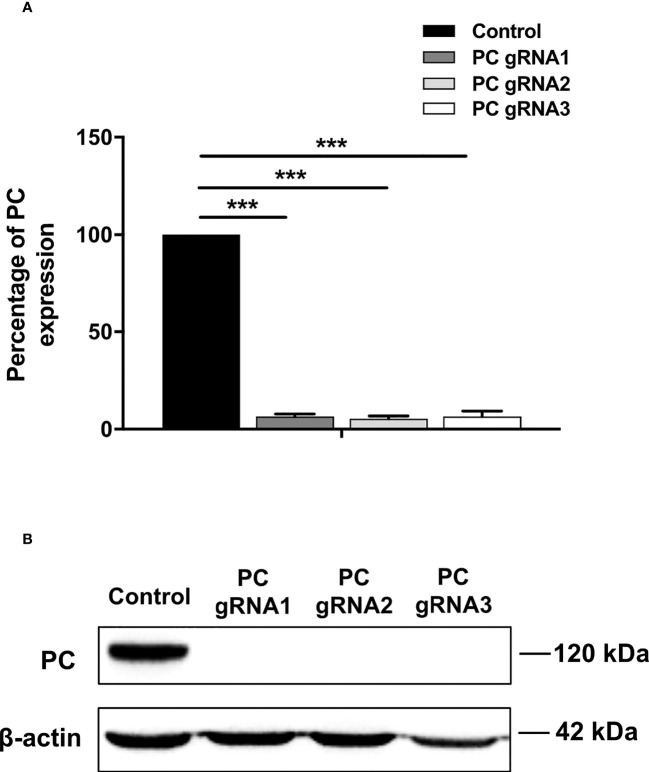
Expression of PC mRNA and protein in PC KO cell lines. **(A)** Q-PCR analysis of PC mRNA. The PC/18s rRNA expression of PC KO cells was relative to that of HT-29-transfected with empty vector (control), which was arbitrarily set to 100%. The results were obtained from three independent experiments, and the statistical analysis was conducted using ANOVA where ***p < 0.001. **(B)** Representative Western blot analysis of PC expression in three PC KO HT-29 clones (PC-gRNA1, PC-gRNA2, and PC-gRNA3), compared to HT-29 cells transfected with empty vector (control). Western blot was performed from three independent experiments.

**Figure 2:**
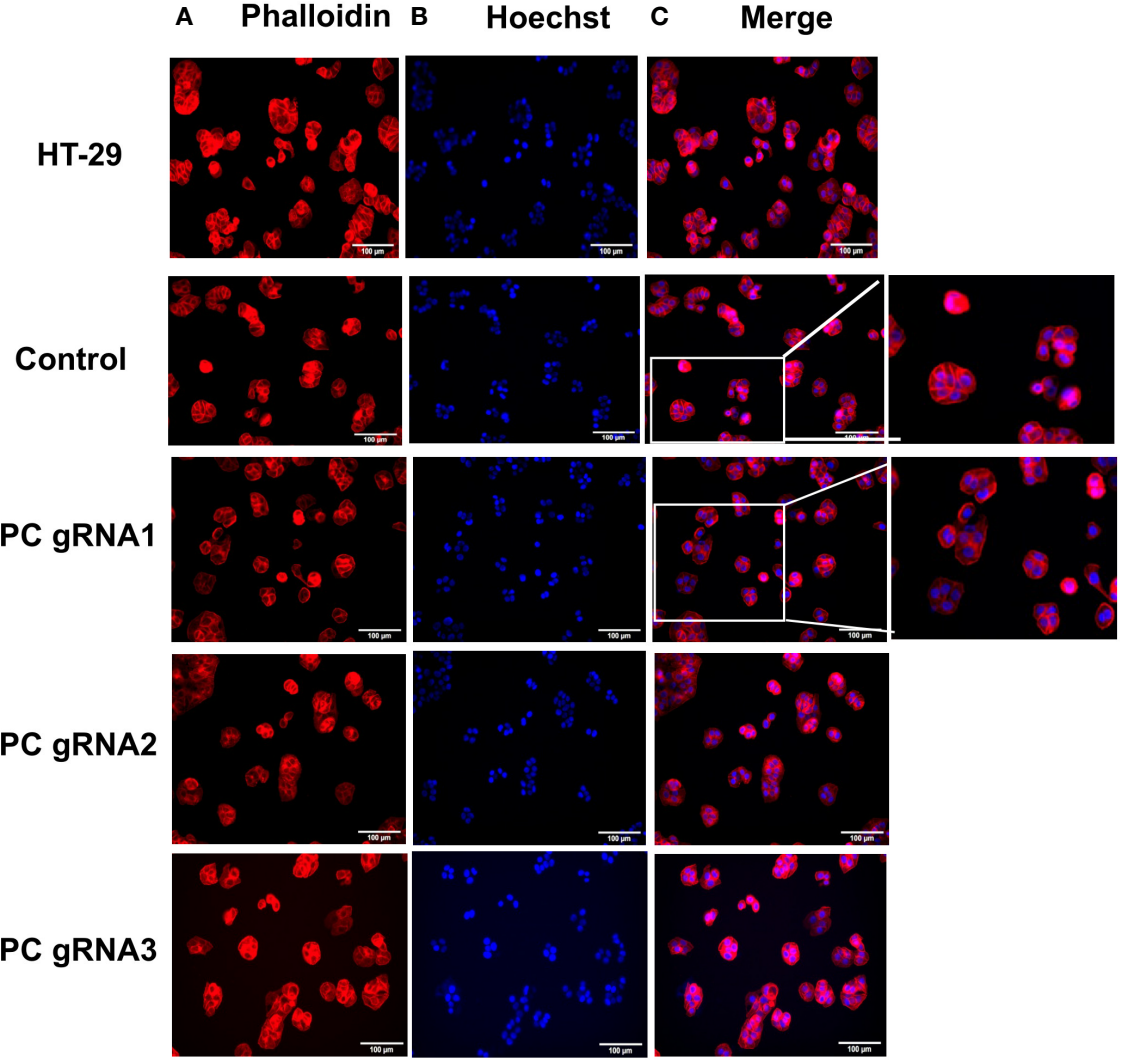
Morphology of PC KO HT-29 cell lines. Immunofluorescent images of parental HT-29, HT-29-empty vector (control) and three PC KO cells (gRNA1, gRNA2, and gRNA3) stained with phalloidin **(A)** and Hoechst 3342 **(B)** and overlay of phalloidin and Hoechst images **(C)**.

Three stable PC KO HT-29 cell lines, namely PC-gRNA1, PC-gRNA2, and PC-gRNA3, were randomly selected and expanded for further characterization. All PC KO clones had remaining PC mRNA less than 2% of the cells transfected with empty vector (control) (**Figure 1A**). Western blot analysis of these three PC KO clones revealed an undetectable level of PC protein (**Figure 1B**). Examination of the cellular structure of PC KO cells by staining cytoplasmic and nucleus with phalloidin and Hoechst 33342, respectively, showed indistinguishable morphology from HT-29 cell line transfected with empty vector (control) and parental HT-29 cell line (**Figure 2**).

**Figure 3:**
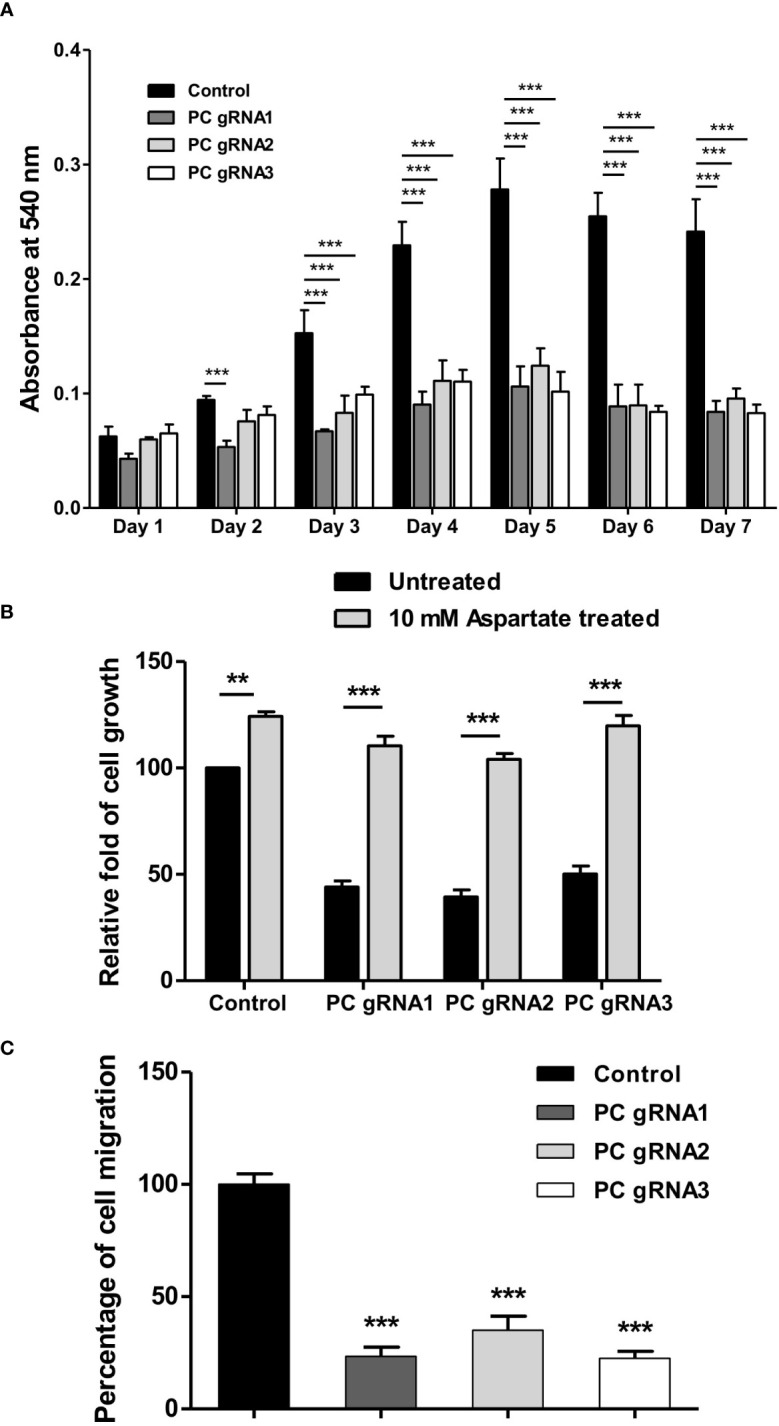
Impaired growth and cell migration of PC KO HT-29-cells. **(A)** Growth rate of PC KO HT-29 cells assessed by MTS assay over 7 days. **(B)** Restoration of growth of PC KO HT-29 cell lines with 10 mM aspartate for 48 h. **(C)** Percentage of migration ability of PC KO HT-29 cells relative to HT-29 transfected with empty vector (control), which was arbitrarily set to 100%. Cells were allowed to migrate through transwell over 48 h. The results were obtained from three independent experiments, and the statistical analysis was conducted using one-way ANOVA where **p < 0.01, ***p < 0.001.

To examine whether ablation of PC expression affects the growth of HT-29 cells, growth phenotypes of PC KO HT-29 cells were assessed over seven days by MTS assay. As shown in **Figure 3A**, all three PC KO clones started to show growth inhibition 25-40% lower than the control cell line on day 2 and became 50%-60% lower throughout the whole time-course. To examine whether supplementation of PC’s reaction product can restore growth of PC KO cells, we performed rescue experiment using aspartate rather than oxaloacetate because oxaloacetate cannot pass through plasma membrane. Once inside cell, aspartate can readily be converted to oxaloacetate by transamination reaction. As shown in **Figure 3B**, 10 mM aspartate was able to restore growth of PC KO cell lines to the similar level as that of the parental HT-29 cell line, suggesting that the growth deficiency is associated with the depletion of PC’s reaction product.

Furthermore, the growth inhibition of all PC KO clones was associated with a 60-70% reduction in their ability to migrate to transwell (**Figure 3C**). These results indicate that PC was essential for the growth and migration of HT-29 cells.

### PC KO induces apoptosis

**Figure 4:**
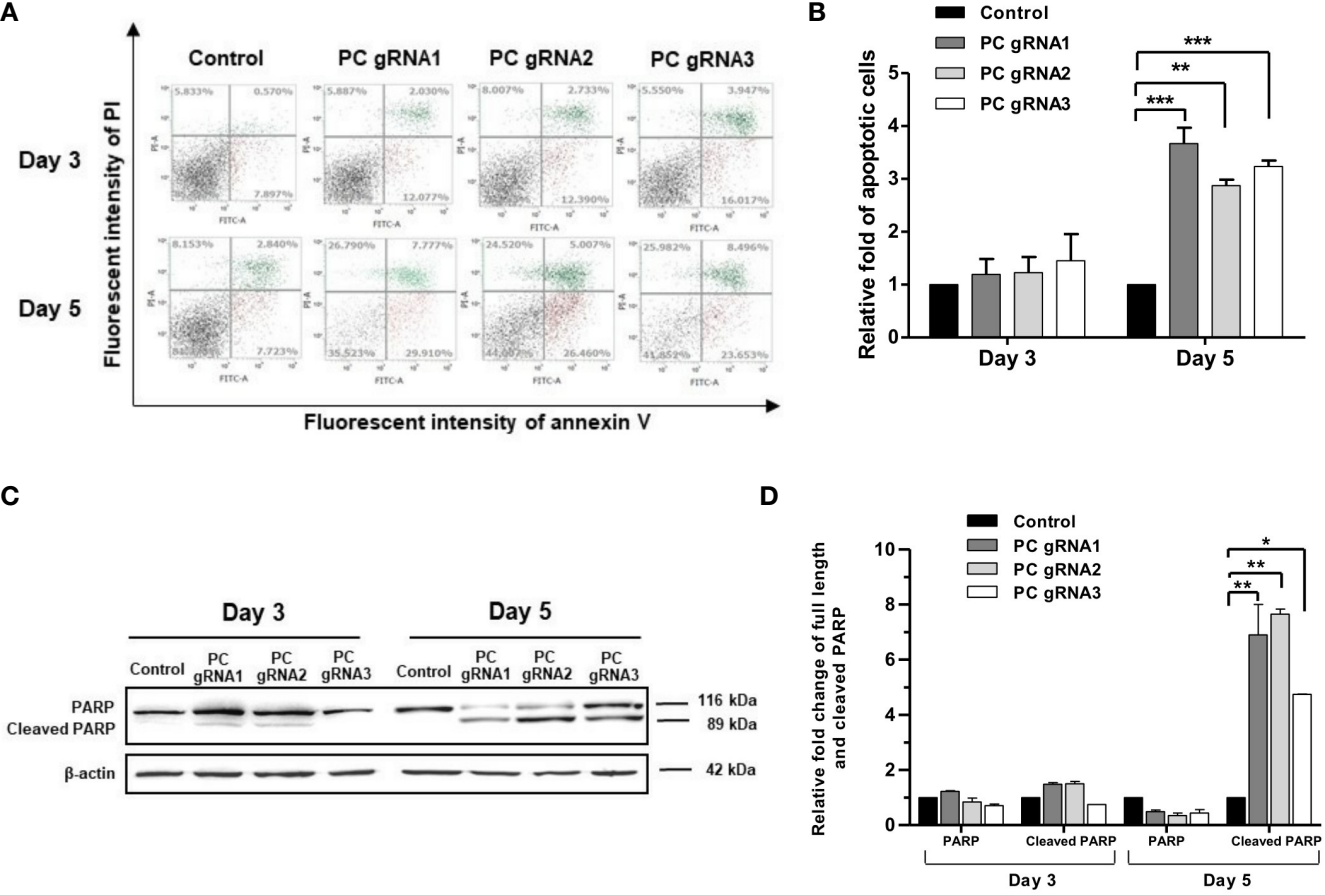
Apoptotic induction in PC KO HT-29 cells. **(A)** Distribution of cell population of PC KO HT-29 cells in live (bottom left), early apoptosis (bottom right), late apoptosis (top right) and dead (top left) at days 3 and 5, respectively. Y-axis is the fluorescent intensity of propidium iodide (PI) which stains DNA and X-axis is the fluorescent intensity of annexin V, an apoptotic marker. **(B)** Relative fold change of apoptotic cells is shown as means ± SD from three independent experiments. **(C)** Representative Western immunoblot of full-length and cleaved PARP in each PC KO HT-29 cell line at days 3 and 5. **(D)** Relative abundance of full length and cleaved PARP bands after normalizing with β-actin. The relative full length and cleaved PARP bands in each PC KO clone were relative to that of HT-29 cells transfected with an empty vector (control), which was arbitrarily set to 1 at each time point. *p <0.05, **p < 0.01, ***p < 0.001.

Activation of apoptosis was also confirmed by detecting poly (ADP-ribose) polymerase (PARP) cleavage. **Figure 4A** shows the apoptosis profiles of PC KO clones at days 3 and 5 by flow cytometry. The fold change of apoptotic cells of the PC KO clones in relative to control is shown in **Figure 4B**. **Figure 4C** shows the representative Western blot analysis of PARP cleavage in all PC KO clones. There was no significant change in the full-length PARP on day 3. However, the level of full-length PARP was decreased by 50-60%, accompanied by a 5-7-fold increase of cleaved PARP at day 5 in all three PC KO clones (**Figure 4D**), consistent with their apoptotic profiles in **Figure 4A**. This result indicates that apoptosis induction is at least partly responsible for the growth retardation of PC KO HT-29 cells.

### Down-regulation of key lipogenic enzymes in PC KO HT-29 cells

**Figure 5:**
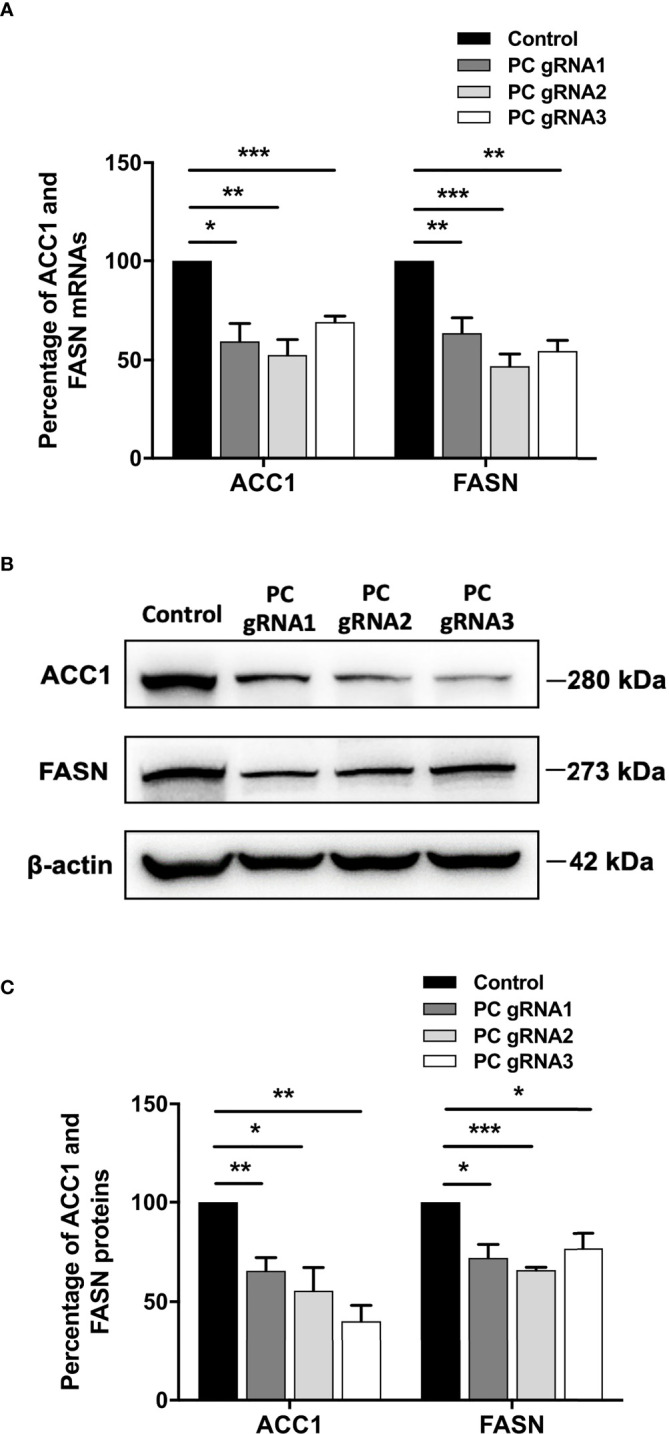
Down-regulation of ACC1 and FASN in PC KO HT-29 cells. **(A)** Percentage of ACC1 and FASN mRNA expression in each PC KO clone compared to that of control HT-29 cell line. **(B)** Representative Western blots of ACC1 and FASN. **(C)** Percentage of ACC1 and FASN protein expression quantitated from **(B)**. The results were obtained from three independent experiments. *p < 0.05, **p < 0.01 and ***p < 0.001.

*De novo* lipogenesis is a crucial anabolic pathway in several types of cancers. Because PC supports *de novo* lipogenesis by providing oxaloacetate, which is combined with acetyl-CoA to form citrate (24–26), ablation of PC expression can potentially disrupt the supply of oxaloacetate, thereby affecting *de novo* lipogenesis. As the acetyl-CoA carboxylase 1 (ACC1) and fatty acid synthase (FASN) are the two regulatory enzymes of *de novo* lipogenesis, we measured their mRNA and protein levels by qPCR and Western blot analysis, respectively. All PC KO clones had both ACC1 and FASN mRNA levels 40-60% lower than the control cell line (**Figure 5A**). The decrease of both ACC1 and FASN mRNAs was accompanied by 50% and 35% down-regulation of their proteins, respectively (**Figures 5B, C**).

### PC KO cells are more sensitive to 5-fluorouracil and glutaminase inhibitor

Because mitochondrial anaplerosis consists of both pyruvate carboxylation and glutaminolysis, PC KO cells may switch to using glutaminolysis to support their growth. The glutaminase inhibitor, Telaglenastat (CB-839) (27) was used to inhibit the growth of PC KO cells. In addition, we also evaluated the sensitivity of PC KO cells to the 5-fluorouracil (5-FU), the standard first-line chemotherapeutic drug for colorectal cancer (28).

**Figure 6:**
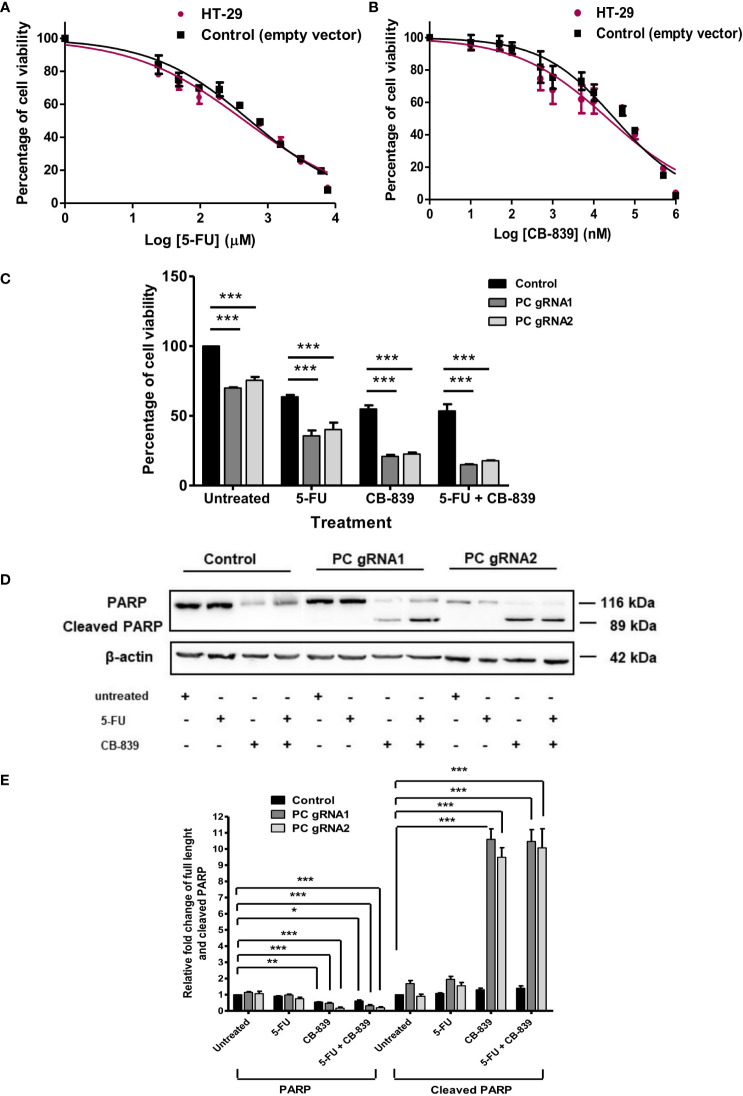
CB-839 but not 5-FU increases its cytotoxicity and induces apoptosis of PC KO HT-29 cells. Cytotoxicity of 5-FU **(A)** or CB-839 **(B)** on parental HT-29 cells and HT-29 cells transfected with empty vector (control) after 48 h exposure to each drug. The viability of cells exposed to each concentration of drug was relative to that of cells exposed to no drug, which is arbitrarily set as 100%. The means ± SD of three independent experiments are plotted. IC_50_ of each drug was calculated from the graph. **(C)** Percentage of the viability of the PC KO HT-29 cells following 48 h exposure to CB-839 or 5-FU at their IC_50_ relative to the control cell line which was arbitrarily set to 100%. **(D)** Representative Western blot analysis of full length and cleaved PARP of PC KO cells following 48 h exposure to 5-FU or CB-839 alone or both. **(E)** Relative abundance of full length PARP and cleaved PARP bands in PC KO HT-29 cells exposed to 5-FU, CB-839 or 5-FU+ CB-839 after normalization with actin. The abundance of full length PARP and cleaved PARP bands in each PC KO clones were relative to the control HT-29 cell line, which was arbitrarily set to 1. The results were obtained from three independent experiments. *p < 0.05, **p < 0.01 and ***p < 0.001.

**Figures 6A, B** showed the cytotoxicity of 5-FU and CB-839 at various concentrations on parental HT-29 and empty-vector transfected HT-29 cell lines after 48 h exposure to the drug, respectively. In general, CB-839 inhibited the growth of both control cell lines in a dose-dependent manner with its half-maximal inhibitory concentration (IC_50_) of approximately 65 μM. Similar inhibition was observed with cells grown in the presence of different concentrations of 5-FU. The IC_50_ of 5-FU for both control cell lines was also similar (4 mM) (**Figure 6A**). We then used this IC_50_ of both drugs to evaluate sensitivity to PC KO cells. In the absence of a drug, the growth of PC KO cell lines was already 20-40% lower than that of the control cell line (**Figure 6C**). In the presence of CB-839, both PC KO cell lines showed 60-70% further inhibition of growth compared to the control cell line, indicating a significant contribution of the glutaminolysis to support HT-29 cells when PC is absent. In the presence of 5-FU, the growth of PC KO cell lines was further inhibited by 40-50%, while the combination of CB-839 and 5-FU did not further inhibit the growth of control cell line but further inhibited growth of both PC KO cell lines. This result indicates that PC KO cells are more vulnerable to combined drug than the control cell line (**Figure 6C**).

The increased sensitivity of PC KO cells to CB-839 and 5-FU suggests that this may be mediated through apoptotic induction. Western analysis of PARP cleavage, an indication of apoptotic induction, was performed, as shown in **Figure 6D**. Control and PC KO cell lines treated with 5-FU did not show the apparent change of PARP cleavage; however, both PC KO cell lines exposed to CB-839 showed 9-10-fold induction of PARP cleavage, while the control cell line did not show a significant change of cleaved PARP. A combination of CB-839 and 5-FU did not further increase PARP cleavage compared with those treated with CB-839 alone (**Figure 6E**). These data indicate that PC KO is prone to CB-839-induced apoptosis. The lack of full-length PARP and cleaved PARP changes in both control and PC KO cell lines treated with 5-FU suggests that the cytotoxic effect of this drug may not be mediated through apoptosis. In line with our result, Metzig MO et al. (29) found that 5-FU could inhibit CRC growth *via* other non-apoptotic cell death, including tumor necrosis factor-α-induced necroptosis (29, 30).

## Discussion

Increased biosynthesis is one of the key metabolic reprogramming, enabling cancer cells to synthesize enough structural components for the new cancer cells. Anaplerosis is an essential biochemical pathway that feeds carbon skeletons into the TCA cycle to support the biosynthesis of amino acids, structural lipids, and nucleic acids. Several previous studies showed that PC is overexpressed in many types of cancer where it supports growth and metastasis. Our previous study showed that PC is overexpressed in colon tissues of CRC patients, suggesting its crucial role in supporting the growth and invasion of CRC. Using the HT-29 colon cell line to represent a highly invasive CRC cell line *in vivo* (31), we showed that PC KO HT-29 cell lines displayed a marked reduction of proliferation throughout the whole time-course of the assay. This phenotypic defect was similarly reported in PC-deficient cancers such as glioblastoma (18), MDA-MB-231 breast cancer (16), non-small cell lung cancer (14), papilloma thyroid carcinoma (19, 32), and ovarian cancer (33). The marked increase of apoptotic cells and PARP cleavage indicates that apoptosis is at least in part responsible for the growth inhibition of PC KO HT-29 cells. The decision of cells to continue or discontinue is tightly regulated through the availability of nutrients and key cellular metabolites, known as metabolic checkpoints, at various phases of the cell cycle (34, 35). Several metabolic enzymes can regulate cell fate decisions through their products. Altered levels of these metabolites can create metabolic stress through the induction of p53 and the Bcl-2 family (36). Depletion of mitochondrial oxaloacetate as a result of PC KO can likely affect its downstream products such as aspartate, nucleotides and lipids. Depletion of these biomolecules at metabolic checkpoints, in turn, triggers apoptosis (34). Unlike the PC-deficient MDA-MB-231 cells, which displayed G2/M cell cycle arrest before apoptosis (37), we did not see the apparent disruption of a specific phase of the cell cycle (data not shown).

A marked reduction of migration, a pre-requisite step of invasion and metastasis, observed in the PC KO HT-29 cell lines supports our previous finding that high PC expression in colon cancer tissue is associated with perineural invasion and lymph node metastasis (20). The marked inhibition of cell growth is likely attributed to the perturbation of TCA cycle activity through oxaloacetate depletion. Because the direct transamination of amino acids to oxaloacetate produces aspartate, an important carbon and nitrogen donor for *de novo* synthesis of nucleotides, lack of PC would reduce the aspartate pool for nucleotide biosynthesis (14, 38). Furthermore, depletion of oxaloacetate would also reduce its conversion to citrate, which is an important precursor of *de novo* lipogenesis. Because cancer cells rely exclusively on *de novo* lipogenesis to produce membrane lipids, depletion of PC would disrupt the biosynthesis of these lipids and restrict cancer cell growth. Phannasil et al. (38) showed that PC-deficient MDA-MB-231 cells showed a marked reduction of palmitate, a major composition of fatty acids in the cell membrane. We also found that PC KO HT-29 cells showed down-regulation of both ACC1 and FASN, two key regulatory enzymes of *de novo* lipogenesis. Down-regulation of these two enzymes can potentially reduce *de novo* lipogenesis of the cells. Compelling evidence indicates that the motility of cancer cells requires lipid rafts, which are made up of specific sphingolipids and cholesterol. The decrease in lipid synthesis can potentially affect these structures, limiting the motility of cancer cells (39, 40). Liu et al. (32) have recently reported that the PC knockdown-papillary thyroid cancer lines, PTC1, also showed a marked decrease of cell motility accompanied by reduced fatty acid synthesis and down-regulation of expression of FASN but not ACC1. Although the reduction of FASN expression was associated with decreased expression of SREBP1c, a transcriptional regulator of the FASN gene, it is still unclear how depletion of oxaloacetate results in down-regulation of SREBP1c expression in PC knockdown PTC1 cells. Nevertheless, the defective phenotypes of growth and migration of PC KO HT-29 and PC-knockdown PTC1 cell lines appear to share a common defect in decreased expression of key lipogenic enzymes. The exact molecular mechanism linking down-regulation of both ACC1 and FASN in PC KO HT-29 cells warrants further investigations.

**Figure 7:**
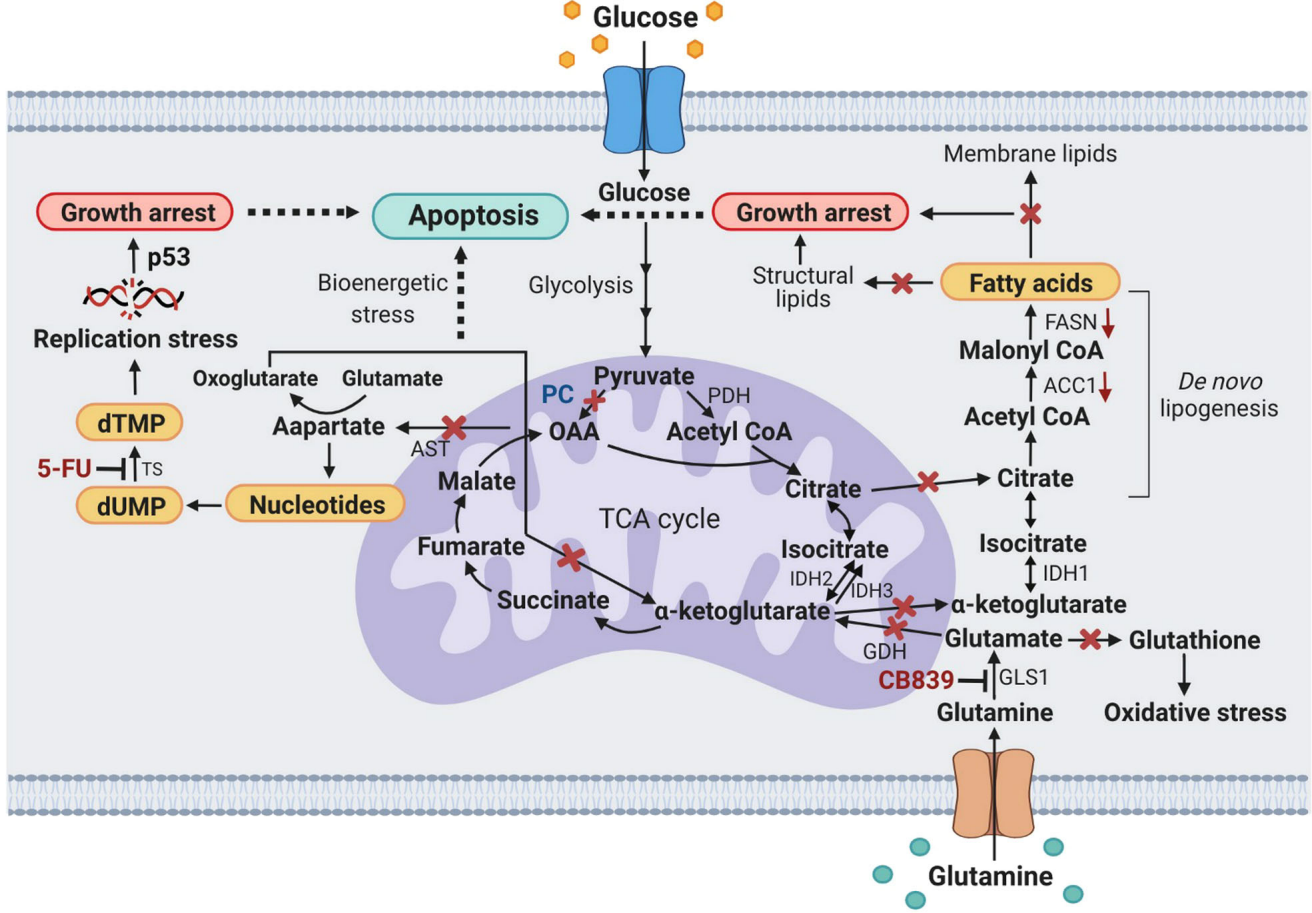
A schematic diagram showing metabolic stress-induced apoptosis in PC KO HT29 cells exposed to CB-839 or 5-FU. X, inhibition or perturbation of pathway; (↓) down-regulation. ACC1, acetyl-CoA carboxylase 1; AST, aspartate aminotransferase; dTMP, deoxythymidine monophosphate; dUMP, deoxyuridine monophosphate; FASN, fatty acid synthase; GDH, glutamate dehydrogenase; GLS1, glutaminase 1; IDH1, 2, 3, isocitrate dehydrogenase 1, 2, 3; OAA, oxaloacetate; PC, pyruvate carboxylase; p53, tumor protein 53; PDH, pyruvate dehydrogenase; TS, thymidylate synthase.

Glutaminolysis is another anaplerotic reaction that can function independently from pyruvate carboxylation. CB-839 is one of the chemotherapeutic drugs which has been under clinical trials for many types of cancer, including colorectal cancer (41). We found that in the presence of CB-839 at its IC_50_, the growth of PC KO HT-29 cells was further inhibited more than that of the control HT-29 cell line, indicating that glutaminolysis is still active in PC KO HT-29 cells. In line with our study, Cheng et al. (18) showed that the PC knockdown glioblastoma cell line did not display a marked growth inhibition under glutamine-rich conditions. However, the PC KO glioblastoma cells showed a dramatic growth restriction under glutamine depletion conditions, indicating that inhibition of one anaplerotic reaction induces a compensatory activation of another reaction. It is noted that growth inhibition of PC KO HT-29 cells after 48 h exposure to CB-839 was more pronounced than HT-29 control cell line. Furthermore, CB-839 also induced more PARP cleavage in PC KO HT-29 cells, suggesting that the simultaneous blockage of both PC and glutaminolysis by CB-839 probably introduces more metabolic stress to the cells. CB-839 is known to cause cell death by perturbing the TCA cycle, which in turn activates the mitochondrial apoptotic pathway (42), increases sensitivity to mTORC1 inhibition (43), and accretion of mitochondrial reactive oxygen species-induced apoptosis (44). Inhibition of both PC and glutaminolysis may abrogate the anaplerotic input of carbons into the TCA cycle, thereby affecting TCA cycle activity. Such perturbation can induce metabolic stress and make cells more sensitive to apoptosis. A recent study showed that sufficient levels of cytosolic aspartate availability determine cell survival when glutaminolysis is compromised. Alkan et al. (45) showed that inhibition of aspartate export through ablation of the mitochondrial aspartate-glutamate carrier 1 gene in C1C12 cancer cells causes them to be more sensitive to apoptosis under CB-839 treatment. According to this scheme, aspartate can only be produced directly from oxaloacetate, and PC is the only enzyme that produces oxaloacetate. Although glutamine can contribute to aspartate production through glutaminolysis, further downstream reactions are required before glutamate can be converted to oxaloacetate, as shown in **Figure 7**. Therefore, inhibition of glutaminolysis by CB-839 when PC is absent would dramatically reduce mitochondrial aspartate production and likely limit cytosolic aspartate, similar to that observed for glutamate-aspartate carrier 1 knockout C1C12 cancer cell. A combination of chemotherapeutic drugs with glutaminase inhibitors has recently been exploited to increase the efficacy of cancer treatment (41). For example, Momcilovic et al. (46) showed that in combination with CB-839, erlotinib induces metabolic crisis and cell death, resulting in rapid regression of non-small cell lung cancer. Thompson et al. (47) showed that CB-839 enhances carfilzomib-induced endoplasmic reticulum stress and apoptosis in multiple myeloma cells.

5-FU is the most frequently used chemotherapeutic drug for CRC and it inhibits thymidylate synthase, a key enzyme in thymidine synthesis. Inhibition of nucleotide synthesis by 5-FU induces cell death *via* multiple pathways, including increased apoptosis and autophagy, production of reactive oxygen species-induced oxidative damage, in many types of cancer, including colorectal cancer (48, 49). We found that PC KO HT-29 cells are also more sensitive to 5-FU, albeit the percentage of growth inhibition was less than that of CB-839. However, 5-FU does not seem to increase PARP cleavage in PC KO HT-29 as that of CB-839 cells, suggesting that the synergistic action on cell death of PC KO may be different from that of CB-839.

Recently, two small molecules, namely, ZY-444 (50) and Erianin (51) have been reported to inhibit PC activity. They also possess a very low IC_50_ within sub-micromolar range and can inhibit growth of many types of cancer including breast, ovarian, lung, colorectal, lung and hepatocellular cancers with minimal effect on normal cells. Similar to our result, exposure of these cancer cell lines to ZY-444 or Erianin also induces apoptosis. It will be interesting to see whether inhibition of PC by these two inhibitors also affects expression of key lipogenic enzymes and increases sensitivity to 5-FU and CB-839 as observed in our study. The reports on these two PC inhibitors also mark a crucial step toward further investigations of their anticancer activity in preclinical and clinical studies.

**Figure 7** proposes the metabolic stress-induced apoptosis in PC KO HT-29 cells exposed to CB-839 and 5-FU. Ablation of PC expression in HT-29 cells and inhibition of glutaminase by CB-839 block anaplerotic input of carbons from pyruvate carboxylation and glutaminolysis to the TCA cycle, which can perturb its activity, leading to bioenergetic stress. Depletion of oxaloacetate and α-ketoglutarate (oxoglutarate) as a result of PC KO and CB-839 treatment would also perturb the citrate supply toward the *de novo* lipogenesis. This effect somewhat causes the downregulation of ACC1 and FASN and reduces cellular fatty acids, resulting in growth arrest. Inhibition of GLS1 would also reduce glutamate, an important precursor for glutathione, causing PC KO cells to be prone to oxidative stress. Ablation of PC expression would also reduce oxaloacetate, resulting in depletion of the aspartate pool available for nucleotide synthesis. Simultaneous inhibition of thymidylate synthase by 5-FU would result in replication stress, inducing apoptosis.

In summary, we showed that ablation of PC expression in HT-29 cells inhibits growth, and migration, induces apoptosis and markedly affects the expression of two critical lipogenic enzymes. PC KO HT-29 cells are also more sensitive to chemotherapeutic drugs, CB-839 and 5-FU. Our study highlights PC’s crucial role in supporting colon cancer cells’ survival under both basal and exposure to chemotherapeutic drugs.

## Data availability statement

Further inquiries regarding original data can be directed to the corresponding author.

## Author contributions

JN: performed, designed experiments, analyzed data, and wrote the first draft of the manuscript; SSi: performed experiments and analyzed data; SSa: performed experiments and analyzed data; KV: supervision and resources; SJ: designed and supervised the experiments, edited manuscript, administration and funding. All authors contributed to the article and approved the submitted version.

## Funding

This work was supported by the International Research Network grant (IRN59W0003) from the Thailand Science Research and Innovation (TSRI) to SJ. SSi was supported by the Science Achievement Scholarship of Thailand (SAST). SSa was supported by PhD scholarship under IRN59W0003 research grant program.

## Conflict of interest

The authors declare that the research was conducted in the absence of any commercial or financial relationships that could be construed as a potential conflict of interest.

## Publisher’s note

All claims expressed in this article are solely those of the authors and do not necessarily represent those of their affiliated organizations, or those of the publisher, the editors and the reviewers. Any product that may be evaluated in this article, or claim that may be made by its manufacturer, is not guaranteed or endorsed by the publisher.
